# Preparation of a Nanosized As_2_O_3_/Mn_0.5_Zn_0.5_Fe_2_O_4_ Complex and Its Anti-Tumor Effect on Hepatocellular Carcinoma Cells

**DOI:** 10.3390/s90907058

**Published:** 2009-09-04

**Authors:** Jia Zhang, Dongsheng Zhang

**Affiliations:** 1 School of Basic Medical Science, Southeast University, Nanjing 210009, China; 2 School of Clinical Medical Science, Southeast University, Nanjing 210009, China;E-Mail: happyzhj2008@yahoo.com.cn

**Keywords:** As_2_O_3_/Mn_0.5_Zn_0.5_Fe_2_O_4_ complex, preparation, anti-tumor, hepatocellular carcinoma cells

## Abstract

Manganese-zinc-ferrite nanoparticles (Mn_0.5_Zn_0.5_Fe_2_O_4_, MZF-NPs) prepared by an improved co-precipitation method and were characterized by transmission electron microscopy (TEM), X-ray diffraction (XRD) and energy dispersive spectrometry (EDS). Then thermodynamic testing of various doses of MZF-NPs was performed *in vitro.* The cytotoxicity of the Mn_0.5_Zn_0.5_Fe_2_O_4_ nanoparticles *in vitro* was tested by the MTT assay. A nanosized As_2_O_3_/Mn_0.5_Zn_0.5_Fe_2_O_4_ complex was made by an impregnation process. The complex’s shape, component, envelop rate and release rate of As_2_O_3_ were measured by SEM, EDS and atom fluorescence spectrometry, respectively. The therapeutic effect of nanosized As_2_O_3_/Mn_0.5_Zn_0.5_Fe_2_O_4_ complex combined with magnetic fluid hyperthermia (MFH) on human hepatocelluar cells were evaluated *in vitro* by an MTT assay and flow cytometry. The results indicated that Mn_0.5_Zn_0.5_Fe_2_O_4_ and nanosized As_2_O_3_/Mn_0.5_Zn_0.5_Fe_2_O_4_ complex were both prepared successfully. The Mn_0.5_Zn_0.5_Fe_2_O_4_ nanoparticles had powerful absorption capabilities in a high-frequency alternating electromagnetic field, and had strong magnetic responsiveness. Moreover, Mn_0.5_Zn_0.5_Fe_2_O_4_ didn’t show cytotoxicity *in vitro*. The therapeutic result reveals that the nanosized As_2_O_3_/Mn_0.5_Zn_0.5_Fe_2_O_4_ complex can significantly inhibit the growth of hepatoma carcinoma cells.

## 1. Introduction

Current hyperthermia treatment strategies centre around the objective of preferential killing of malignant cells and localized heating of tissue deep inside the patient. Magnetic fluid hyperthermia (MFH) offers a means of doing this, which involves direct intratumoral injection of magnetic fluids into the target region, and then the particles are selectively heated in an externally applied alternating magnetic field (AMF). The technique uses the Curie temperature (Tc) of the magnetic material in magnetic response heating to achieve automatic temperature control and a constant temperature. Thus this technique could avoid overheating of the tissue. *In vitro* and *in vivo* experiments with magnetic fluids have documented significant antitumor effects in a murine model of liver cancer [1]. Heat also enhances the effectiveness of radiotherapy and magnifies the cytotoxicity of many anticancer drugs. For this reason, hyperthermic treatment, alone or in combination with traditional anticancer treatments, is receiving a great deal of attention.

Arsenic trioxide (As_2_O_3_), a kind of Traditional Chinese Medicine, has drawn researchers’ great interest due to its high efficacy in the treatment of acute promyelocytic leukaemia (APL). It also has been tested in some solid cancers, such as hepatocellular carcinoma [2], gastric carcinoma [3], breast cancer [4], etc. After intravenous or oral administration [5], As_2_O_3_ has been accompanied a series of side effects, such as skin reactions, gastrointestinal upset, hepatitis and even cardiotoxicity [6]. To make the best use of the drug and reduce the harmfulness to the body, it is very important to find a new form of As_2_O_3_ for clinical therapy.

In this study, As_2_O_3_ was integrated with Mn_0.5_Zn_0.5_Fe_2_O_4_ nanoparticles, which have super-paramagnetic characteristics. This feature is quite suitable for use in hyperthermia. On the one hand, a certain concentration of magnetic nanoparticles could absorb high power and transform it into heat in an alternating magnetic field, while barely damaging peripheric tissue. If used *in vivo*, the magnetic nanoparticles could be inducted in the target region by an external magnetic field. In this way, not only does the drug level in the tumor region rise, but also the drug dose decreases. Finally the nanosized As_2_O_3_/Mn_0.5_Zn_0.5_Fe_2_O_4_ complex could produce chemotherapy and thermotherapy effects at the same time.

## 2. Results and Discussion

### 2.1. Characteristics of Mn_0.5_Zn_0.5_Fe_2_O_4_ Nanoparticles

**Figure 1.**
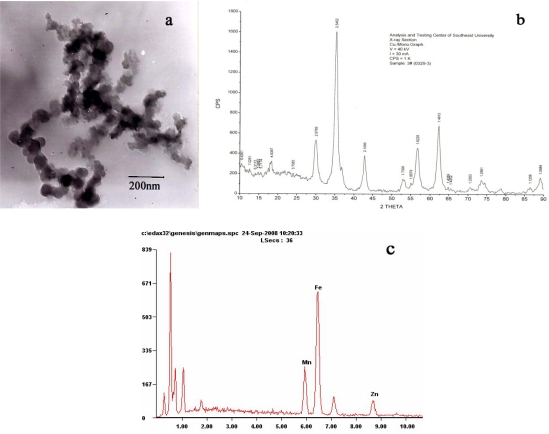
Characterization of Mn_0.5_Zn_0.5_Fe_2_O_4_. (a) TEM image of the nanosized Mn_0.5_Zn_0.5_Fe_2_O_4_. (b) XRD of Mn_0.5_Zn_0.5_Fe_2_O_4_. (c) EDS of Mn_0.5_Zn_0.5_Fe_2_O_4_.

**Figure 2.**
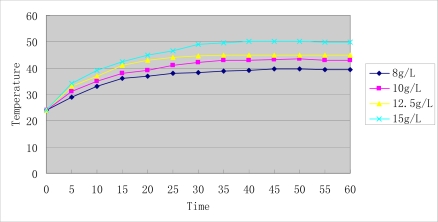
Heating test curve of sized Mn_0.5_Zn_0.5_Fe_2_O_4_ nanoparticle fluid.

Figure 1a shows an image of Mn_0.5_Zn_0.5_Fe_2_O_4_ nanoparticles acquired by TEM. It shows that they were nearly spherical, with high electron-density and uniform in size. The X-ray diffraction pattern of the ferrite sample is shown in Figure 1b. The observed diffraction lines were found to correspond to those of a standard manganese ferrite pattern, thereby indicating that the samples have spinel structure. The particle size of the samples had been estimated from the broadening of the X-ray diffraction peaks, using the Scherrer equation for Lorentzian peak: d = 0.9 λ/(w−w_1_)/cosθ. The average particle size was found to be between 6 and 22 nm. EDS reports showed At% of Mn, Zn, Fe were 17.58%, 16.44% and 65.98%, respectively, which is consistent with a molar ratio of 0.5:0.5:1. Figure 2 shows the results of thermodynamic test of various doses of Mn_0.5_Zn_0.5_Fe_2_O_4_ nanoparticles fluid. The nanoparticles were dispersed in 0.9% NaCl and exposed to a high-frequency alternating electromagnetic field (output current equal to 30 A) for 60 min. As the concentrations of magnetic fluid (MF) increase, its temperature rose from 39.5 to 50 °C, and the temperature was stable after exposure to the magnetic field for 40 minutes. Our study thus shows that Mn_0.5_Zn_0.5_Fe_2_O_4_ nanoparticles have powerful absorption capabilities in a high-frequency alternating electromagnetic field and strong magnetic responsiveness.

### 2.2. Cytotoxicity of Mn_0.5_Zn_0.5_Fe_2_O_4_ Nanoparticles

**Figure 3.**
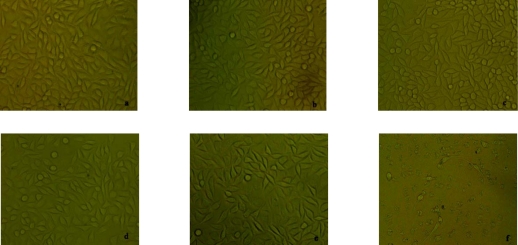
Pictures of cytotoxicity evaluation on L929 cells, which were cultured in different concentrations of Mn_0.5_Zn_0.5_Fe_2_O_4_ leaching liquor. (a) Negative group. (b) 25% Leaching liquor. (c) 50% Leaching liquor. (d) 75% Leaching liquor. (e) 100% Leaching liquor. (f) Positive group.

The morphological changes of L929 cells after treatment with different concentrations of Mn_0.5_Zn_0.5_Fe_2_O_4_ leaching liquor were observed by inverted microscopy. As shown in Figure 3, the shapes and growth of the treated cells were similar to that of cells in the negative group. They exhibited normal features, such as clear edges, homogeneous staining and no cell fragments, while the cells of the positive group became small and globular, and even parts of cells were suspended 48 hours later. Only small amounts of cells survived. The results of the MTT assay are shown in Table 1. According to RGR and toxicity grade conversion table (see Section 3.3), the toxicity of Mn_0.5_Zn_0.5_Fe_2_O_4_ leaching liquor was classified as grade 1, which was safe to the cells. This is in agreement with the findings from inverted microscopy and demonstrated that Mn_0.5_Zn_0.5_Fe_2_O_4_ didn’t show cytotoxicity *in vitro*.

### 2.3. Characterization of the Nanosized As_2_O_3_/Mn_0.5_Zn_0.5_Fe_2_O_4_ Complex

**Figure 4.**
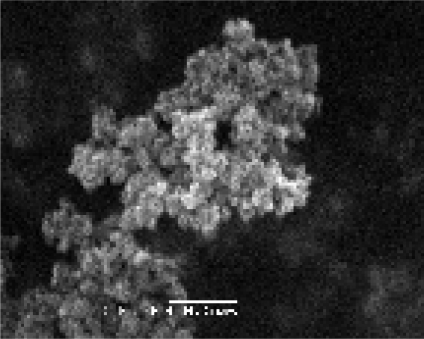
SEM image of the nanosizedAs_2_O_3_/Mn_0.5_Zn_0.5_Fe_2_O_4_.

**Figure 5.**
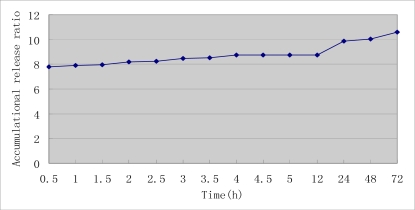
The accumulative release rate of As_2_O_3_/Mn_0.5_Zn_0.5_Fe_2_O_4_ nanoparticles.

The self-prepared nanosized As_2_O_3_/Mn_0.5_Zn_0.5_Fe_2_O_4_ complex particles are approximately spherical and uniform in size (Figure 4). The EDS result confirmed that the prepared complex only contained As, Mn, Zn, Fe and O. The envelop rate of As_2_O_3_ was 0.260%. The release rate of As_2_O_3_ is shown in Figure 5. As time elapsed, the release of as increased gradually.

### 2.4. Inhibition of HepG2 Proliferation after Treated with Nanosized Complex Combined with MFH

The therapeutic results of the MTT assay are shown in Table 1. The cell growth inhibitory ratio (IR) of the As_2_O_3_ alone group, the Mn_0.5_Zn_0.5_Fe_2_O_4_ magnetic nanoparticles group and the nanosized As_2_O_3_/Mn_0.5_Zn_0.5_Fe_2_O_4_ complex group were 32.3%, 30.7% and 55.2%, respectively. The negative control group and the experimental groups had a great difference (*p* < 0.05). Due to the combined advantages of the thermotherapy and the chemotherapy, the nanosized As_2_O_3_/Mn_0.5_Zn_0.5_Fe_2_O_4_ complex in combination with MFH could more significantly inhibit the proliferation of the HepG2 cells compared to the As_2_O_3_ alone group and Mn_0.5_Zn_0.5_Fe_2_O_4_ magnetic nanoparticles in combination with MFH group (*p* < 0.05). So we may conclude that the therapeutic effect of nanosized As_2_O_3_/Mn_0.5_Zn_0.5_Fe_2_O_4_ complex in combination with MFH on HepG2 cells is much better than that of As_2_O_3_ alone or Mn_0.5_Zn_0.5_Fe_2_O_4_ in combination with MFH.

### 2.5. Nanosized As_2_O_3_/Mn_0.5_Zn_0.5_Fe_2_O_4_ Complex Combined with MFH Induces Apoptosis of HepG2 Cells

The present study disclosed that the combination of thermotherapy and chemotherapy resulted in synergistic inhibition of hepatoma [7]. Both As_2_O_3_ [8] and ferromagnetic fluid thermotherapy [9] could induce apoptosis of HepG2 cells.

**Figure 6.**
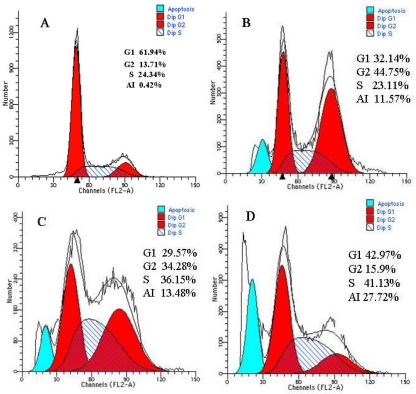
Apoptosis of HepG2 cells induced by the different methods. A) The negative control group. the cells of G2 phase account for 13.71.%, the apoptotic rate was 0.42%. B) The single As2O3 group the cells of G2 phase account for 44.75%, the apoptotic rate was 11.57%. C) The single Mn_0.5_Zn_0.5_Fe_2_O_4_magnetic nanoparticles group. the cells of G2 phase account for 36.15%, the apoptotic rate was 13.4%. D) The nanosized As_2_O_3_/Mn_0.5_Zn_0.5_Fe_2_O_4_ complex group. The cells of G2 phase account for 15.9%, the apoptotic rate was 27.72%.

In our study, the flow cytometry assay showed that the apoptotic indexes of HepG2 cells of the single As_2_O_3_ group, the single Mn_0.5_Zn_0.5_Fe_2_O_4_ magnetic nanoparticles group and the nanosized As_2_O_3_/Mn_0.5_Zn_0.5_Fe_2_O_4_ complex group after treatment were 11.57%, 13.48% and 27.72%, respectively. However, the apoptotic index of the control group was 0.42%. In the experimental groups, we found a significant hypodiploid peak before the G1 phase, which was the apoptotic peak (Figure 6). At the same time, there was a prominent cell cycle blockage in the G2/M phase in these groups. Other studies also found As_2_O_3_ and hyperthermia could arrest the cell cycle at S or G2/M phase in tumor cells respectively [9,10], so As_2_O_3_/Mn_0.5_Zn_0.5_Fe_2_O_4_ in combination with MFH could induce an obvious cell cycle disturbance and apoptosis.

## 3. Instruments and Methods

### 3.1. Preparation and Characterization of Nanosized Mn_0.5_Zn_0.5_Fe_2_O_4_ Magnetic Nanoparticles

Mn-Zn ferrite of composition Mn_0.5_Zn_0.5_Fe_2_O_4_ was prepared by the precipitation method (for details see [6]). Its shape was observed by H-600 transmission electron microscope (TEM). X-ray diffraction (XRD) was used to analyse its crystal structure and diameter. An energy dispersive spectrometer (EDS) was employed to assay its composition.

### 3.2. Heating Test of Nanosized Mn_0.5_Zn_0.5_Fe_2_O_4_ in vitro

Various doses of Mn_0.5_Zn_0.5_Fe_2_O_4_ nanoparticles were dispersed in 5 mL 0.9% NaCl, to concentrations of 8, 10, 12.5 and 15 g/L, respectively. Then the nanoparticles fluids were placed in a flat-bottomed cuvette. This in turn was placed 5 mm from the bottom of the cuvette to the center of hyper-thermia-coil of high frequency electromagnetic field (SP-04C, Shenzhen, China). The output frequency was 230 KHz and the output current was 30 ampere, heating 1 h and the temperature was measured at 5 min intervals.

### 3.3. The cytotoxicity of Mn_0.5_Zn_0.5_Fe_2_O_4_ Nanoparticles

The sterile nanoparticles were diffused in PRPMI 1640 medium (containing 10% fetal calf serum) for 72 h at 37 °C. After centrifugalization, the supernatant was filtered to get leaching liquor of 100% concentration. L929 cells in a 96-well plate, 6,000 cells per well, were treated after 24 hours incubation with various concentrations of leaching liquor (100%, 75%, 50% and 25% leaching liquor), and incubation was continued for 48 hours. At the same time, cells cultured with RPMI1640 medium containing 10% fetal calf serum as the negative control group, and cells with 0.7% polyacrylamide as positive group were also prepared. Inverted microscopy was used to observe general morphological changes of the L929 cells, then the MTT assay was performed and the OD value was measured at 492 nm. The cell relative growth rate was calculated as follows: RGR% = OD of experimental group/OD of control group × 100%. According to the biological evaluation of medical devices (test for in vitro cytotixicity (ISO10993-5:1999, IDT)), the toxicity grade is precise when the experimental results show grade 0 and grade 1. The results show grade 2, which needs evaluate by RGR% and the morphological changes of cultured cells. The toxicity grade is uncertian when it belongs to other grades.

### 3.4. Preparation and Characterization of Nanosized As_2_O_3_/Mn_0.5_Zn_0.5_Fe_2_O_4_ Complex

The nanosized As_2_O_3_/Mn_0.5_Zn_0.5_Fe_2_O_4_ complex was prepared using an impregnation process. As_2_O_3_ was purchased from Sigma (St Louis, MI, U.S.A.). Satis quantum Mn_0.5_Zn_0.5_Fe_2_O_4_ nanoparticles were added into the solution of As_2_O_3_ (0.01 mg/mL, pH = 5, adjusted by acetic acid) under ultrasonic sound dispersion conditions. After 30 min standing at 80 °C, the production (nanosized As_2_O_3_/Mn_0.5_Zn_0.5_Fe_2_O_4_ complex) was centrifuged at 2,000 g/min for 10 min and rinsed twice with absolute alcohol, then dried. Their shape, component and the envelop ratio were measured with SEM, EDS and atom spectrophotometer, respectively. Subsequently the release rate of As_2_O_3_ was measured. One hundred mg of As_2_O_3_/Mn_0.5_Zn_0.5_Fe_2_O_4_ complex were put in a visking bag filter and 50 mL 0.9% NaCl was placed outside as dialysis mediator. The system was agitated at the speed of 50 rpm in 37 °C. In first 5 hours, we sampled every 0.5 h intervals and supplemented the same volume of 0.9% NaCl. Then we sampled at the following 24, 36 and 48 h, respectively. The amount of As_2_O_3_ of the sample was measured by atom fluorescence spectrometry.

### 3.5. The Therapeutic Effects on Cultured Hepatocellular Carcinoma Cells-HepG2

The growth of HepG2 cells treated with nanosized As_2_O_3_/Mn_0.5_Zn_0.5_Fe_2_O_4_ complex combined with MFH was examined by an MTT assay. HepG2 cells were seeded in a 96-well plate with 6,000 cells per well, and after 24 hours incubation they were divided into 4 groups: (1) the negative control group (RPMI1640 medium containing 10% fetal calf serum); (2) the As_2_O_3_ alone group (5 μM of As_2_O_3_); (3) the Mn_0.5_Zn_0.5_Fe_2_O_4_ magnetic nanoparticles combined with MFH group (10 mg/mL); (4) the nanosized As_2_O_3_/Mn_0.5_Zn_0.5_Fe_2_O_4_ complex combined with MFH group (the quantity of As_2_O_3_/Mn_0.5_Zn_0.5_Fe_2_O_4_ and Mn_0.5_Zn_0.5_Fe_2_O_4_ was adjusted to 10 mg/mL of Mn_0.5_Zn_0.5_Fe_2_O_4_ and 5 μM of As_2_O_3_). Each group contained eight wells. The magnetic nanoparticles group and the nanosized As_2_O_3_/Mn_0.5_Zn_0.5_Fe_2_O_4_ complex group were treated with MFH for 60 minutes under a high frequency alternating electro-magnetic field (f = 230 KHz, I = 30 A). Then incubation of all groups was continued for 48 hours, the MTT assay was performed and OD value was measured at 492 nm. The cell growth inhibition rate was calculated as follows: (1-OD of experimental group / OD of control group)×100%.

### 3.6. Flow Cytometry Assay

The cells of control group and experimental groups were collected and rinsed in 0.1 PBS (pH 7.2–7.4) three times, resuspended and fixed in 70% ethanol at 4 °C overnight. Cells were centrifuged and resuspended in 0.1 g/L RNase A at 37 °C for 30 min and in 0.05 g/L propidium iodide at 4 °C for 30 min. The cell cycle was analyzed by flow cytometer (FACS Vantage SE, Becton-Dickson Co).

## 4. Conclusions

Manganese zinc ferrites (Mn-Zn-Ferrit), a new kind of soft magnetic material, have been widely used in biomedicine applications including magnetic resonance, imaging contrast enhancement, tissue specific release of therapeutic agents, hyperthermia, and so on. Here, they are chosen for MFH according to their high sensitivity of magnetization to temperature and low Tc. From the results of characterization of TEM, XRD and EDS, it proved Mn_0.5_Zn_0.5_Fe_2_O_4_ were successfully prepared through an improved coprecipitation process. The prepared Mn_0.5_Zn_0.5_Fe_2_O_4_ magnetic nanoparticles had powerful absorption capabilities in the high frequency alternating magnetic field, rising to a steady temperature and showing strong magnetic responsiveness. Mn_0.5_Zn_0.5_Fe_2_O_4_ magnetic nanoparticles also show good biocompatibility, which proved the prepared nanopaticles could be applied in biomedicine.

As_2_O_3_ is a valuable therapeutic tool in hepatoma treatment. Its actions may include its ability to induce cellular apoptosis [11–15], inhibit tumor metastasis [16], decrease the expression of vascular endothelial growth factor (VEGF) [17,18], enhance immune function [19], and so on. On the other hand it lacks targeting ability, and produces some adverse reactions. This limits its application in solid cancers. To resolve the problems, a nanosized As_2_O_3_/Mn_0.5_Zn_0.5_Fe_2_O_4_ complex of As_2_O_3_ has also been prepared. From this study, nanosized As_2_O_3_/Mn_0.5_Zn_0.5_Fe_2_O_4_ complex can be successfully prepared through an impregnation process and As_2_O_3_ can be released gradually from this As_2_O_3_/Mn_0.5_Zn_0.5_Fe_2_O_4_ complex.

The thermochemotherapy of nanosized As_2_O_3_/Mn_0.5_Zn_0.5_Fe_2_O_4_ complex can significantly inhibit the proliferation of cultured hepatocellular cancer cells (HepG2) and induced apoptosis and cell cycle arrest when combined with magnetic fluid hyperthermia. This indicates that hyperthermia and chemotherapy could play a synergistic role. The mechanisms may be: 1) heat could make drug absorption increase due to the addition of blood flow in tumor region; 2) heat could decrease the tumor vessel regeneration for the reduction of synthesis and secretion of VEGF; 3) heat could enhance the cytotoxic effects of the chemotherapeutic drug. We have not confirmed these details, but this study may provide a new method for hepatocellular cancer therapy. However, a lot of work needs to be done if it is to be applied in clinical treatment.

## Figures and Tables

**Table 1.** The results of cytotoxicity of Mn_0.5_Zn_0.5_Fe_2_O_4_ nanoparticles evaluated by MTT assay(*X̄* ± s, n = 8).

**Groups**	**Optical Density (OD)**	**RGR/%**	**Toxicity Grade**
Negative control group	1.035 ± 0.042	100	0
25% Mn_0.5_Zn_0.5_Fe_2_O_4_ leaching liquor	1.017 ± 0.038	98.3	1
50% Mn_0.5_Zn_0.5_Fe_2_O_4_ leaching liquor	1.006 ± 0.051	97.2	1
75% Mn_0.5_Zn_0.5_Fe_2_O_4_ leaching liquor	0.973 ± 0.042	94.0	1
100% Mn_0.5_Zn_0.5_Fe_2_O_4_ leaching liquor	0.952 ± 0.034	91.9	1
Positive group	0.229 ± 0.025	22.1	4

**Table 2.** Growth inhibitory rate of As_2_O_3_/Mn_0.5_Zn_0.5_Fe_2_O_4_ nanoparticles with MFH on HepG2 cells.

**Groups**	**Optical density (OD)**	**Inhibitory rate (%)**

Negative control group	1.207 ± 0.071	0
single As_2_O_3_group (5μm/L)	0.817 ± 0.024	32.3% (1)
single Mn_0.5_Zn_0.5_Fe_2_O_4_ group (10g/L)	0.836 ± 0.034	30.7% (1)
As_2_O_3_/ Mn_0.5_Zn_0.5_Fe_2_O_4_ complex group	0.540 ± 0.037	55.2% (1)(2)

Comparison of experimental groups with negative control group;

(1) *p* < 0.05.

Comparison of nanosized As_2_O_3_/Mn_0.5_Zn_0.5_Fe_2_O_4_ complex group with single As_2_O_3_ group and single Mn_0.5_Zn_0.5_Fe_2_O_4_ magnetic nanoparticles combined with MFH group;

(2) *p* < 0.05.

**Table 3.** The table of RGR and toxicity grade.

**Toxicity grade**	**Relative growth rate (RGR%)**
Grade 0	≥ 100
Grade 1	75–99
Grade 2	50–74
Grade 3	25–49
Grade 4	1–24
Grade 5	0
